# Streamflow classification by employing various machine learning models for peninsular Malaysia

**DOI:** 10.1038/s41598-023-41735-9

**Published:** 2023-09-04

**Authors:** Nouar AlDahoul, Mhd Adel Momo, K. L. Chong, Ali Najah Ahmed, Yuk Feng Huang, Mohsen Sherif, Ahmed El-Shafie

**Affiliations:** 1 Computer Science, New York University Abu Dhabi, Abu Dhabi, United Arab Emirates; 2 Fleet Management Systems & Technologies, Istanbul, Turkey; 3 Faculty of Engineering & Quantity Surveying, INTI International University (INTI-IU), Persiaran Perdana BBN, Putra Nilai, 71800 Nilai, Negeri Sembilan Malaysia; 4 Department of Civil Engineering, College of Engineering, Universiti Tenaga Nasional, 43000 Kajang, Selangor Malaysia; 5 Institute of Energy Infrastructure (IEI), Universiti Tenaga Nasional (UNITEN), 43000 Kajang, Selangor Malaysia; 6 Department of Civil Engineering, Lee Kong Chian Faculty of Engineering and Science, Universiti Tunku Abdul Rahman, Jalan Sg. Long, Bandar Sg. Long, 43000 Kajang, Selangor Malaysia; 7 National Water and Energy Center, United Arab Emirates University, P.O. Box 15551, Al Ain, United Arab Emirates; 8 Civil and Environmental Eng. Dept, College of Engineering, United Arab Emirates University, 15551 Al Ain, United Arab Emirates; 9 Department of Civil Engineering, Faculty of Engineering, University of Malaya (UM), 50603 Kuala Lumpur, Malaysia

**Keywords:** Hydrology, Engineering

## Abstract

Due to excessive streamflow (SF), Peninsular Malaysia has historically experienced floods and droughts. Forecasting streamflow to mitigate municipal and environmental damage is therefore crucial. Streamflow prediction has been extensively demonstrated in the literature to estimate the continuous values of streamflow level. Prediction of continuous values of streamflow is not necessary in several applications and at the same time it is very challenging task because of uncertainty. A streamflow category prediction is more advantageous for addressing the uncertainty in numerical point forecasting, considering that its predictions are linked to a propensity to belong to the pre-defined classes. Here, we formulate streamflow prediction as a time series classification with discrete ranges of values, each representing a class to classify streamflow into five or ten, respectively, using machine learning approaches in various rivers in Malaysia. The findings reveal that several models, specifically LSTM, outperform others in predicting the following n-time steps of streamflow because LSTM is able to learn the mapping between streamflow time series of 2 or 3 days ahead more than support vector machine (SVM) and gradient boosting (GB). LSTM produces higher F1 score in various rivers (by 5% in Johor, 2% in Kelantan and Melaka and Selangor, 4% in Perlis) in 2 days ahead scenario. Furthermore, the ensemble stacking of the SVM and GB achieves high performance in terms of F1 score and quadratic weighted kappa. Ensemble stacking gives 3% higher F1 score in Perak river compared to SVM and gradient boosting.

## Introduction

More people are being harmed by floods worldwide. According to UNISDR^1^, flooding is the primary cause of natural disasters globally, accounting for 90% of all catastrophes. Rivers in Malaysia exhibit significant seasonality, with most peak flows concentrating on torrential rains from the monsoon in the north-east and south-west due to their closeness to the equator^2,3^. Consequently, consistent rain causes rivers to overflow their banks, which causes a considerable volume of streamflow to pass through^4^. Malaysia has had several large floods throughout its history, most notably the worst floods ever in 2006 and 2007, which resulted in significant losses for the government and total economic devastation^5^. The recent rapid population growth within the river's basin diminishes river capacity and accelerates streamflow, increasing flood amplitude and duration^6^. These factors, along with climate changes, has further substantial the frequent occurrence of flood in Malaysia^7^. A simple and low-cost tool for monitoring flood occurrence is streamflow time series monitoring, an effective indicator of trends and changes in the hydro-climatic system^8,9^.

In a generic machine learning context, time series analysis may theoretically be viewed as either a classification or a regression situation. Machine learning streamflow regression has been the most often studied topic in streamflow predicting research^10,11^. Hydrologists often distinguish this form of prediction as numerical forecasting in streamflow regression tasks, where they generate a single-point estimate of its expected value. Early in the year, time series forecasting included models like ARIMA and ARIMAX. However, there is substantial evidence that models based on linearity assumption do not provide good forecasts in streamflow forecasting^12^. These models make predictions based on the dataset's correlation through autocorrelation and partial autocorrelation functions. Recognizing that the linear assumption is inadequate for complicated time series forecasting, researchers proposed an artificial neural network (ANN), which functions as a universal approximation function^13^. Other often used machine learning algorithms include random forest (RF)^14,15^ and gradient boosting (GB)^16,17^. And when uncertainty is factored in, the predicting process may be quantified using probability forecasting, another form of regression^18^. In practice, the over-fitting problem encountered makes it difficult for machine learning to forecast the continuous value with 100% accuracy^19^. A model that does well on both the training and testing datasets is often favorable in machine learning. In essence, the model gathers enough knowledge about the dataset from the inputs to make a generalized judgment^20^.

Contrastingly, a classification task focuses on classifying the prediction into one of the many predetermined categories^21^. The easiest way to categorize streamflow is as a binary task, where streamflow may either be increased or decreased. The theoretical complexity of the multi-class classification problem is greater than that of the binary task, as streamflow is divided into more than two class labels, necessitating additional decision-making^19,22^. The fact that streamflow classification considers more than simply whether or not the streamflow will change today should be stressed. The predicted streamflow classifications are linked to the likelihood of belonging to each class. However, transitioning a time series regression to a classification need careful planning since categorization entails a forced-choice presumptive decision with discrete, rather than stochastic, outcomes^23^. There are situations in the real world where something is not definite, such as "It will rain today," and categorization them is not the best course of action. Though—a streamflow classification can be beneficial, especially in reservoir operations, where it is sometimes necessary to discretize the storage stage in order to derive the operational rule for optimizing the reservoir system^24^. Recently, an illustration of streamflow classification may be seen in the study by Chong, Huang^25^, where they examined two distinct streamflow machine learning formulations. They discovered that scenario-based streamflow forecasts outperform point forecasts in terms of accuracy. However, they also noted that in the absence of other predictors or data-preprocessing techniques, their findings could be biased in favour of univariate streamflow. Given the constraints imposed by numerical point forecasting, classifying streamflow outputs would necessitate a more thorough analysis and potentially a better decision to develop streamflow forecasting.

Another crucial consideration is the choice of a hydrological model. The advent of machine learning may allow a data-driven model to function better compared to a process-driven model but at the price of the physical interpretation of hydrological processes^26^. The current transition to data-driven modeling may be due to the difficulty in fully comprehending the interactions that underlie the hydrological processes, which limits the efficacy of a process-driven model^27^. Despite the reformulation from regression to classification, we hypothesize that the streamflow time series still retain their temporally ordered structure, characterizing them from other TSCs that do not make any assumptions regarding temporal dependency. Typical classification algorithms are not well adapted to such a task since they do not incorporate the time component^28^. Developing an effective AI model to carry out this classification process is therefore necessary. Deep learning technologies, such as long short-term memory (LSTM), give additional feature extraction capabilities that might be used to supplement classic classifier algorithms' lack of time-dependent components. It may collect time series and memorize long-term associations using the memory storage capabilities of LSTM by applying many gates that regulate the information flow. Such qualities may be seen in a variety of applications where sequential information flow is crucial, including robotic control^29^, handwriting recognition^30^, and even time series prediction^31^.

The format of this paper is as follows: Section "Previous works" introduces the previous works related to this study; In Section "The significance of study", the significance of the study is discussed. Section "Materials and methods" describes the dataset used and demonstrates the machine learning and deep learning algorithms used. Section "Results and discussion" presents the results and discussion; Section “Conclusion and future work” summarises the conclusions and recommendations for future research.

## Previous works

### Probabilistic methods

In case water demand, allocation, and flooding event prediction, several studies have considered probabilistic methods to predict the chance of flood. Monte Carlo techniques have been utilized to estimate the probability of a region being impacted by a cyclone any year^32^. Monte Carlo method was found to be easy to implement and can continuously be improved with more data collected over years.

To respond to emergency cases and sudden rainstorms and flooding, integration of decision makers' emotions, dynamic Bayesian network and Dempster–Shafer (DS) evidence theory was proposed^33^. Bayesian network worked effectively to simulate the dynamic change process. Additionally, the DS evidence theory can reduce the subjectivity of the model in dealing with the uncertainty of the evolution process. Another study was demonstrated to help on “scenario-response" paradigm. The target heavy rain event was studied to examine the intricate evolution of emergency response utilizing a constructed scenario Bayesian network^34^. This network was built by fusing the knowledge meta-theory, scenario evolution and Dempster's rule.

To assess the risk and zone the flood disaster, another study was conducted^35^ to highlight the high-risk areas clarifying the reasons behind the potential hazards. The authors analysed the disaster system theory and established the flood disaster evaluation index system for urban agglomerations.

### Machine Learning methods

Artificial neural networks (ANNs) have been used as a useful soft computing tool to predict future water availability from a catchment in real-world scenario^36^. The utilization of ANN was proposed due to the absence of intensive data, which are required for modelling practices in the context of hydrology. Levenberg–Marquardt ANN was able to give good prediction performance^37^.

Another study compared stacked model that combines random forest and multilayer perceptron through elastic net with bidirectional long short-term memory networks for multiple steps ahead streamflow prediction^38^. It was found that the stacked model outperformed the model based on bidirectional LSTM in many cases in predicting the highest flow rate but it was less accurate in predicting low flow rate. The prediction accuracy of both models decreased by increasing the length of the time series. The stacked model has shorter computation times than the bidirectional LSTM.

The evaluation and comparison between various deep learning models including convolutional neural networks (CNN), long short-term memory (LSTM), and self-attention (SA)-LSTM models, with simple extreme learning machine (ELM) model was demonstrated for monthly streamflow prediction^39^. The experiments targeted to predict an unprecedented hydrologic event such as no-flow events and extreme floods. SA–LSTM model was proved to be an effective streamflow prediction model for extreme events.

Explainable AI with long short-term memory (LSTM) has been explored in the literature to predict the streamflow^40^. In their study, the authors utilized the model's explainability using Shapley additive explanations method (SHAP). It was discovered that LSTM model's explainability in predicting the streamflow was enhanced by the SHAP method.

## The significance of study

Forecasting streamflow lowers the risk of flooding and reservoirs while enhancing the management and planning of water resources. Due to its ability to detect the non-linarites and short- or long-term temporal interrelationship, statistical and machine learning techniques have been applied for streamflow forecasting challenges. However, the machine learning models with multivariate streamflow forecasting may be affected by over-fitting problem and inability to predict exact values of streamflow. To address the aforementioned issue, streamflow categorization approach has been proposed in this study to extract patterns from streamflow data and map these features to specific categories.

Due to the highly non-linear pattern, stochastic nature, and the extremely wide range of the streamflow in the selected rivers as shown in Tables 1 and 2, the water resources management strategy concluded to categorize the streamflow into different classes for each time increment and consider the streamflow class is operational constraints and the major component of the water management policy.

**Table 1:** Total duration for each river from eleven rivers.

River data	Total duration
Johor	1st January 1978 to 31st December 1998
Kedah	1st January 1976 to 31st December 2009
Kelantan	1st January 1980 to 31st December 1997
Melaka	1st January 1979 to 31st December 2004
N9	1st January 1980 to 31st December 1995
Pahang	1st January 1988 to 31st December 2009
Perak	1st January 1977 to 31st December 1995
Perlis	1st January 1986 to 31st December 1995
Selangor	1st January 1976 to 31st December 2001
Terengganu	1st January 1977 to 31st December 1996
WPKL	1st January 2010 to 31st December 2010

**Table 2:** Descriptive data analysis of streamflow for the eleven rivers.

River	Mean (m^3^/s)	Median (m^3^/s)	Mode (m^3^/s)	Std. dev. (m^3^/s)	Min. (m^3^/s)	Max. (m^3^/s)	Count
Johor	40.0	28.7	15.9	43.4	0.5	709.7	7670
Kedah	87.1	58.0	26.0	88.3	3.0	1160.0	12,419
Kelantan	495.7	364.2	509.3	587.1	81.7	9775.1	6575
Melaka	5.8	3.3	1.4	7.8	0.0	119.9	9497
N9	0.5	0.2	0.1	1.7	0.0	65.3	5844
Pahang	683.5	520.6	497.3	540.5	133.0	6285.3	8036
Perak	219.6	212.0	250.0	109.7	19.0	988.0	6939
Perlis	0.7	0.0	0.0	1.6	0.0	23.0	3652
Selangor	53.9	44.0	34.7	36.7	2.3	313.9	9497
Terengganu	124.5	78.2	50.0	185.1	9.4	3178.8	7305
WPKL	0.5	19.7	20.8	9.6	10.3	105.6	365

The motivation of this work is to study the possibility of formulating the streamflow prediction task as a classification problem by dividing streamflow into more than five and ten class labels.

The transfer from regression to classification opens the doors to implement various classification models to predict the levels of streamflow which helps for further decision making.

In light of the above, the current work's goal is to examine how deep learning performs in anticipating the streamflow levels in comparison to other classifier algorithms, namely, GB and SVM. Furthermore, an effective technique, stacking ensemble modelling, was also adopted to enhance the performance of the model. Several metrics were used to assess the performance of ML, including accuracy, precision, recall, F1 score, the area under the score, and Quadratic Weighted kappa (QWK).

## Materials and methods

**Figure 1:**
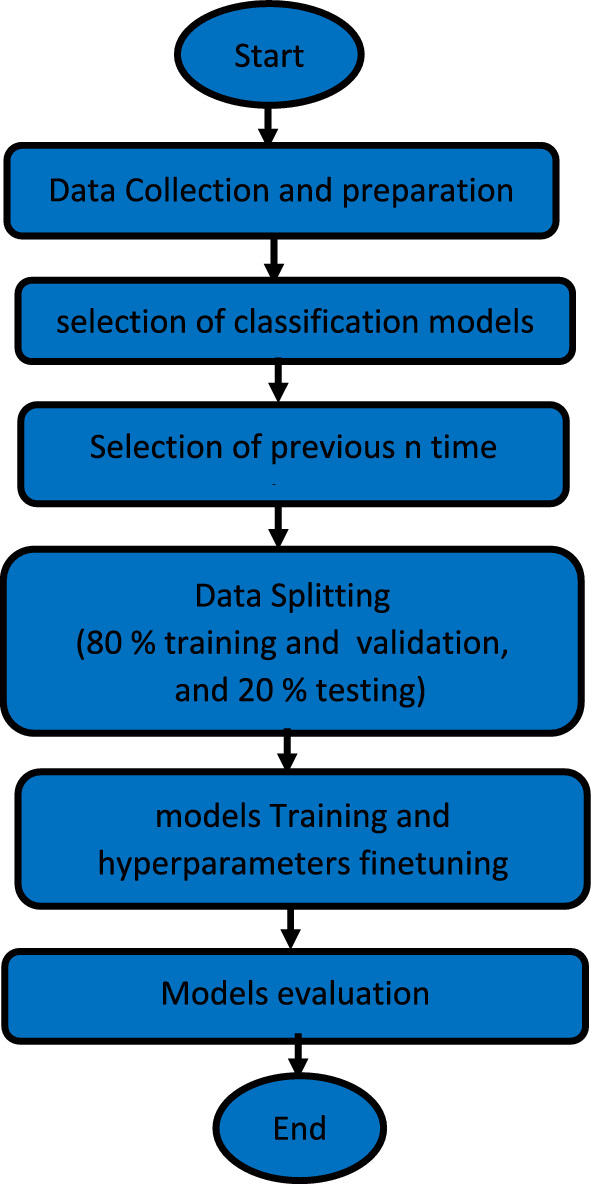
Flow chart of our methodology for streamflow classification using machine learning models.

This section covers the methodology of the presented work, as illustrated in a flow chart in Fig. 1. To begin, we give an overview of data collected from eleven rivers used for flow classification. Second, the methods and classification models used for perdition purposes are detailed with their optimal architectures and hyperparameters.

### Data description

The data used for modelling in this work have daily streamflow values collected for a specific duration, as shown in Table 1. The period of data gathering varies from river to river. Kedah river included the most years of the dataset, with a total of 12,419 sample. In contrast, WPKL had the smallest number of years, with only one year’s worth of data, with only 365 samples. Table 2 shows the basic statistical parameters of the streamflow dataset of each river, which differ in sample size.

**Figure 2:**
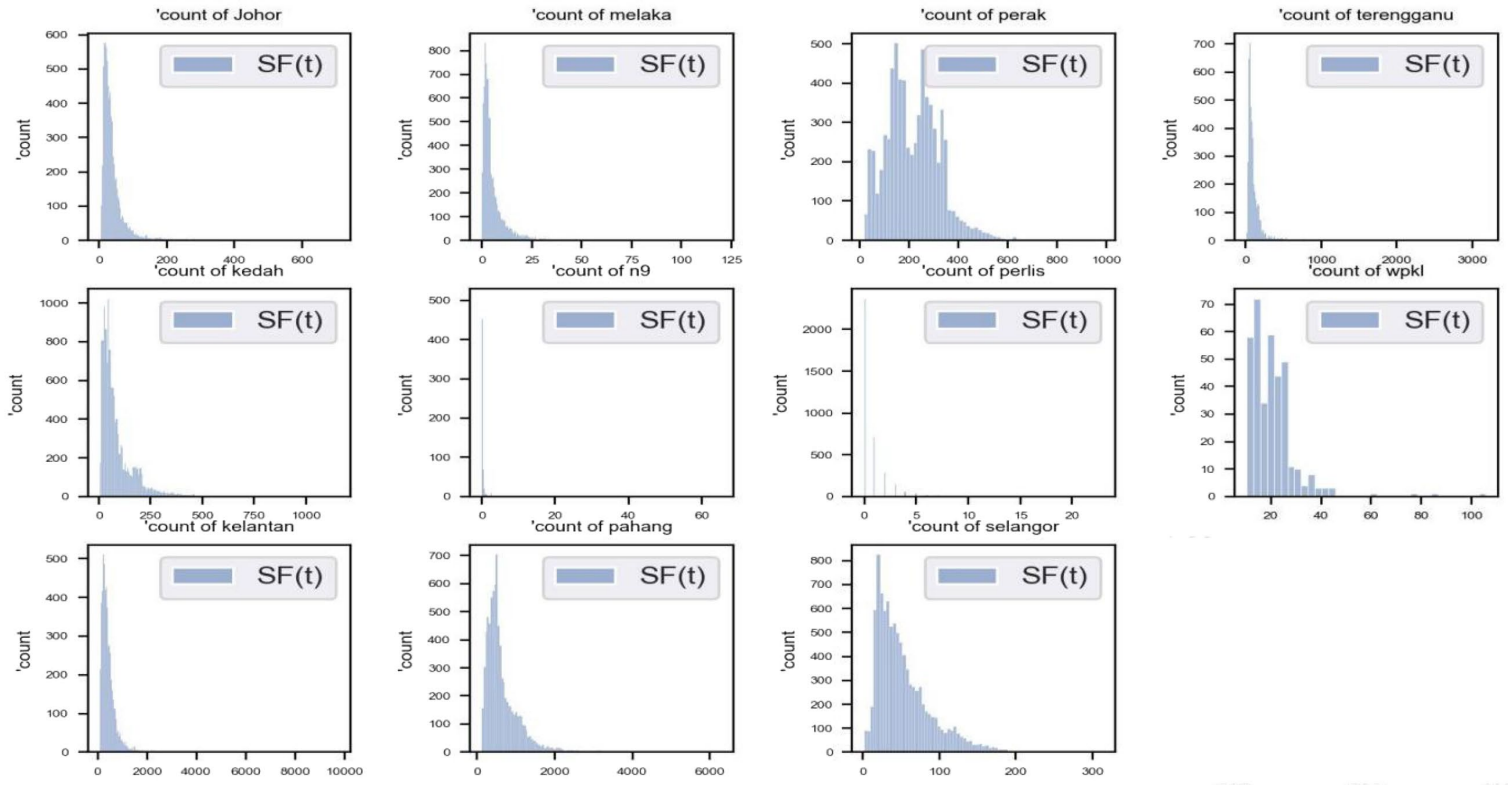
The histogram of streamflow values for eleven rivers.

Figure 2 shows the histogram distribution of streamflow data of each river. As seen in Fig. 2, not all rivers have an identical distribution of streamflow data. The horizontal axis represents the streamflow, and the vertical axis represents the count of the specific range of flow values. The categories (labels) were set according to the range values of streamflow. It is clear that the streamflow samples were abundant in some labels while being scarce in others.

**Figure 3:**
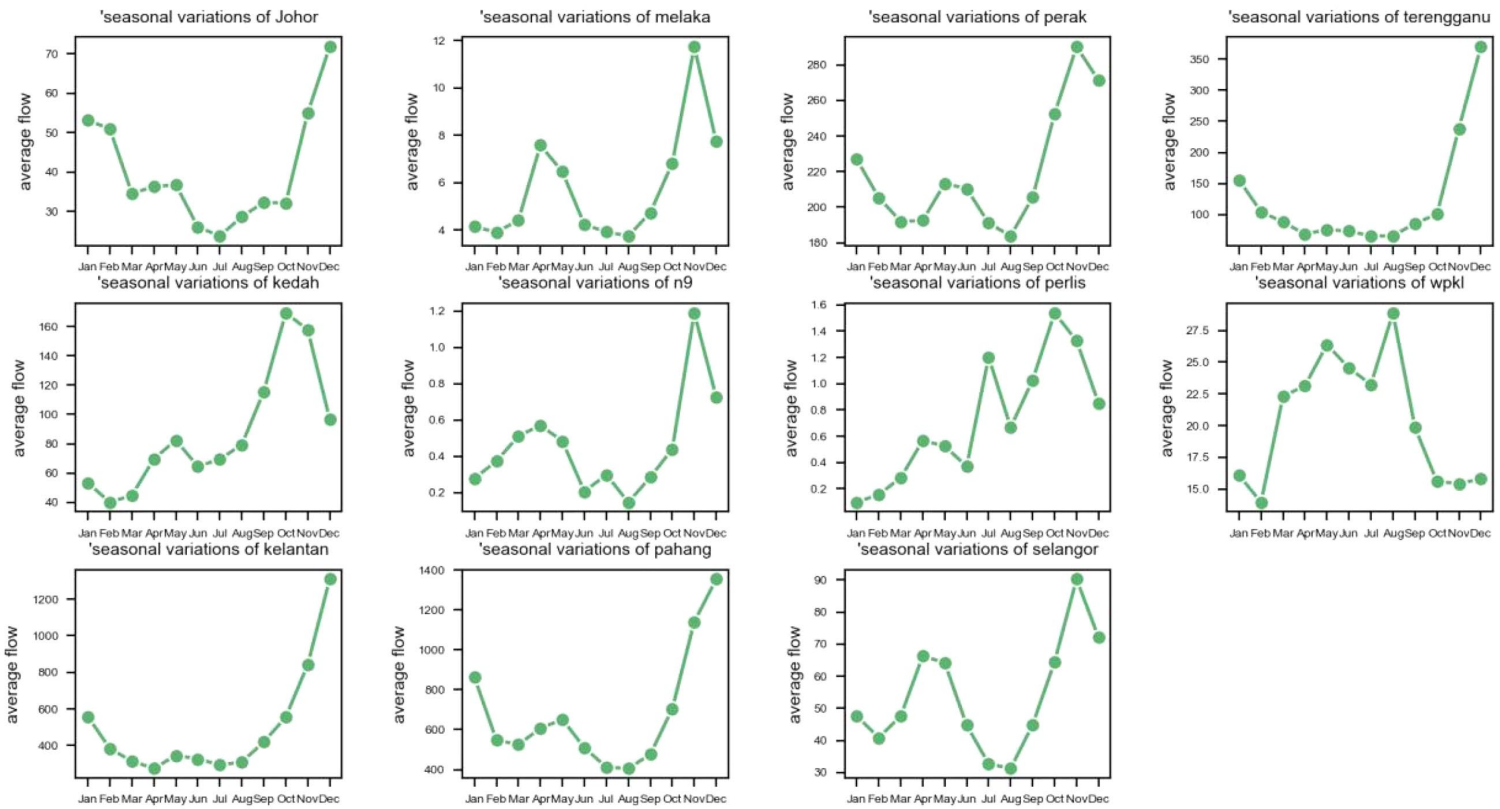
The seasonal variations of streamflow values for eleven rivers.

**Figure 4:**
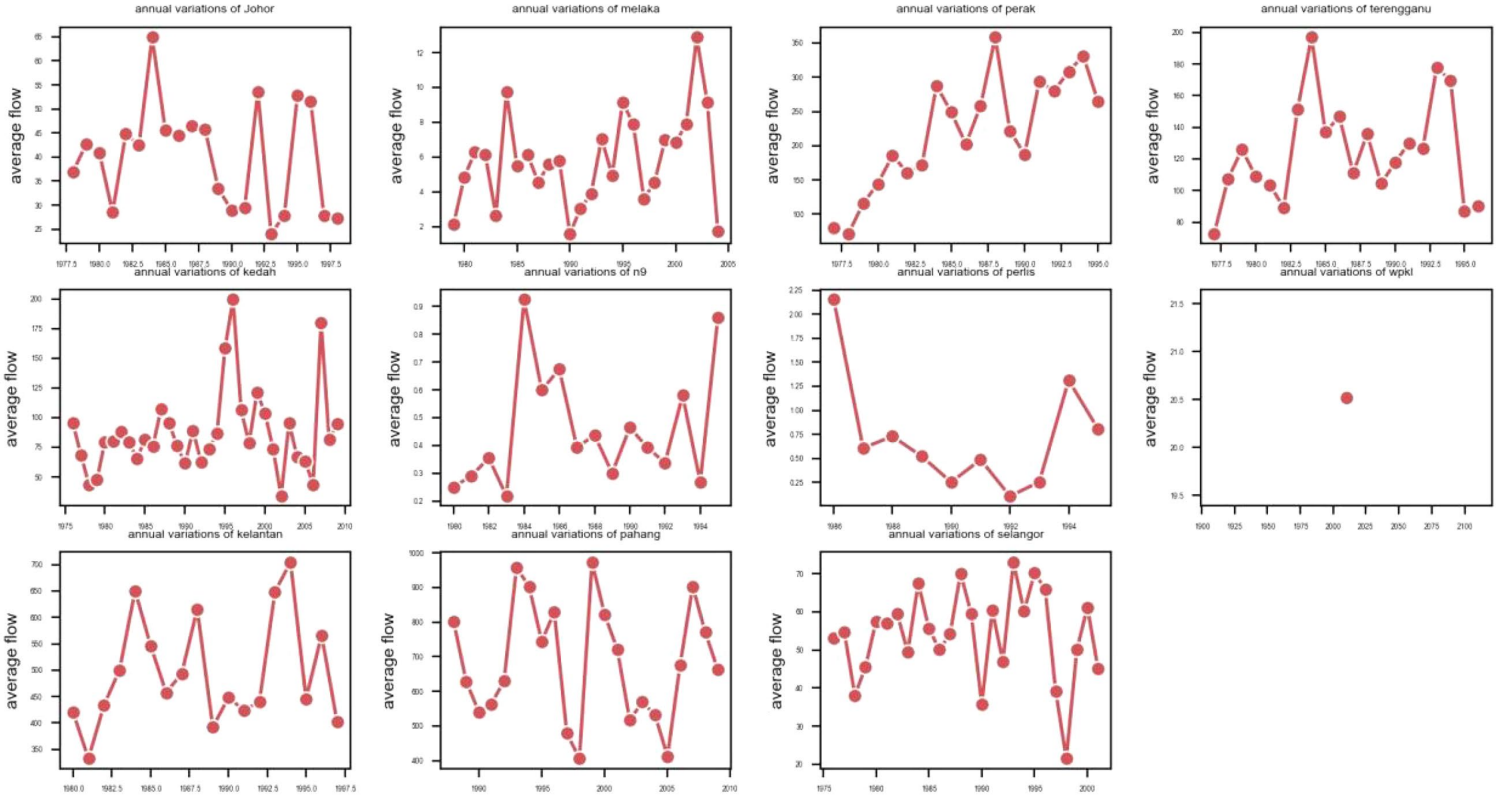
The annual variations of streamflow values for eleven rivers.

Figure 3 depicts seasonal variations in streamflow. We can infer that November and December is when most rivers’ average streamflow are at their peak. Additionally, annual variations of streamflow are shown in Fig. 4. Another characteristic of the data is that the average streamflow of many rivers varies depending on the year. The number of years that have daily data collected is also different from one river to another.

### Data partitioning

This section describes the experimental procedure and data partitioning. The streamflow dataset for models was split into three parts: training, validation, and testing, using a 60%, 20%, and 20% rule, respectively. Parallel to training data, validation data were used to tune the model’s hyperparameters to discover hidden patterns in the input series. It is crucial to have testing data since it allows for the evaluation of generalizability. Finally, the optimized models with the best architecture and hyperparameters were used to evaluate the model for comparison purposes using the testing dataset.

### Feature scaling

MinMax scaler was used to scale the feature vector, including previous n-steps from streamflow time series. This scaler is able to avoid distortion in the data by preserving its shape. Each feature is translated as follows between zero and one as follows:

$$\mathrm{X}\_\mathrm{std }=\frac{(\mathrm{X }-\mathrm{ X}.\mathrm{min}(\mathrm{axis}=0))}{(\mathrm{X}.\mathrm{max}(\mathrm{axis}=0) -\mathrm{ X}.\mathrm{min}(\mathrm{axis}=0))} \#(1)$$

$$\mathrm{X}\_\mathrm{scaled }=\mathrm{ X}\_\mathrm{std }* (\mathrm{max }-\mathrm{ min}) +\mathrm{ min }\#(2)$$

where min, max = feature range.

### Category label annotation

The streamflow was separated into various ranges, with each category generated belonging to one class or label. This paper exhibited two scenarios regarding the number of classes, five and ten. Due to the different characteristics of each river, the modeling required to identify the hidden patterns differ significantly from one another. Tables 3, 4, and 6 show two methods of range division for five and ten categories as follows:

**Table 3:** Data balanced method to divide streamflow range into 5 categories.

Range	R1	R2	R3	R4
Johor	16	24	35.36	54.95
Kedah	26	45	74	137
Kelantan	203.9	292.3	403.4	580.3
Melaka	1.35	2.41	4.07	7.58
N9	0.09	0.14	0.19	0.31
Pahang	362.8	482.7	596.6	954.2
Perak	106	150	188	276
Perlis	0	–	–	1
Selangor	25.15	37.91	53.92	79.07
Terengganu	46.9	65.0	89.1	133.9
WPKL	15.67	20.27	22.5	25.14

**Table 4:** Equal range method to divide streamflow range into 5 categories.

Range	Min	Max	R1	R2	R3	R4
Johor	0.54	536.65	107	214	321	428
Kedah	3	1160	231	462	694	925
Kelantan	81.7	9775.1	1938	3877	5816	7754
Melaka	0.1	119.93	23	47	71	95
N9	0.01	39.13	7	15	23	31
Pahang	133	6285.3	1230	2460	3691	4921
Perak	19	835	163	326	489	652
Perlis	0	16	3	6	9	12
Selangor	8.58	253.75	49	98	147	196
Terengganu	9.4	3178.8	633	1267	1901	2535
WPKL	10.31	105.61	19	38	57	76

#### Data balanced method

This method divided the streamflow into ranges (categories), each with the same number of samples.

#### Equal range method

This method divided the streamflow into ranges using $(\mathrm{maximum }-\mathrm{ minimum})/ 5$ in five-category scenario or $(\mathrm{maximum }-\mathrm{ minimum})/10$ in ten-category scenario to have same length for all caegories.

Table 5 illustrates the algorithm used to formulate the streamflow prediction as a classification problem. This algorithm used ranges available in Tables 3 and 4 for the scenario of five categories utilizing the data balanced and the equal range method. The same algorithm has also been applied in the scenario of ten categories using only the balanced data method, as shown in Table 6.

**Table 5:**
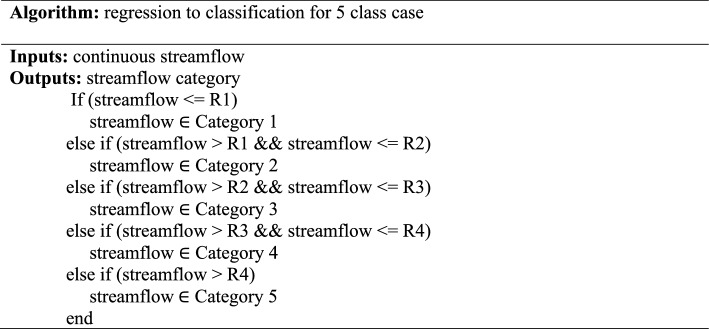
Algorithm to formulate streamflow prediction case as a classification case for five categories.

Statistical significance was stated in the case of *p* < 0.05 and was highlighted by light gray color.

**Table 6:** Balanced data method to divide streamflow range into 10 categories.

Range	R1	R2	R3	R4	R5	R6	R7	R8	R9
Johor	11.92	15.58	19.26	23.58	29.26	35.36	43.68	54.95	81.57
Kedah	18	26	34	45	57	74	98	137	192
Kelantan	159.9	203.9	247.5	292.2	341.9	403.3	483.8	579.0	756.2
Melaka	0.79	1.35	1.83	2.41	3.13	4.07	5.52	7.58	12.05
N9	0.06	0.09	0.11	0.14	0.16	0.19	0.23	0.31	0.57
Pahang	277.4	362.8	423.9	482.7	524.8	596.6	717.3	954.2	1267.1
Perak	62	106	134	150	168	188	226	276	341
Perlis	0	0	0	0	0	0	1	1	2
Selangor	20.55	25.15	31.74	37.91	45.28	53.92	64.66	79.07	103.67
Terengganu	37.7	46.9	55.2	64.9	76.7	89.0	105.2	133.8	201.5
WPKL	12.54	15.67	18.33	20.27	21.03	22.53	24.38	25.2	30.43

### The proposed classification models

This section discusses classification models used in this work to classify streamflow values into five or ten categories, along with the optimized architectures and hyperparameters. The models included Extreme Gradient Boosting (GB), Support Vector Machine, an ensemble stacked of SVM and GB, and Long Short-Term Memory (LSTM). For each model, several experiments were conducted to select the best architecture and hyperparameters. The criteria for evaluation and selection were based on classification performance metrics such as the F1 score and quadratic weighted kappa (QWK).

### Support vector machine (SVM)

The support Vector Machine is one of the models used for the streamflow classification task. SVM is a supervised learning model that can be used for classification tasks. SVM works by separating data vectors at inputs to maximize the margins from these vectors. The transformation is done from a non-linear decision surface to a linear one for a higher number of dimension spaces. SVM offers a number of hyperparameters, including kernel and regularization parameter C. SVM’s kernel is a crucial hyperparameter to turn the inputs into the required form^41^. We tested various linear and non-linear kernel functions such as Gaussian (RBF), sigmoid, and polynomial kernels to select one that produced better results with validation data. We conducted experiments to select a regularization parameter and kernel carefully. These optimal values can generate the best performance indicators, such as F1 score and QWK. SVM using RBF kernel and regularization factor of 100 was determined to deliver the best F1 score.

During SVM training, the hyperplane is selected to enlarge the distance to the nearest vector. The objective is to minimize the loss function, which is as follows:

1 $${min}_{w,b} \frac{1}{2} {w}^{T} w +C \sum_{i=1}\mathrm{max}(0,{y}_{i} ({w}^{T }\phi \left({x}_{i}\right)+b))$$

where *W* is a weight vector*, b* is a bias vector*, ϕ* is the identity function, and *C* is a regularization constant.

Non-linear classifiers result from non-linear kernels by computing the inner-product between two $\phi$ functions as follows :

2 $${K \left({x}_{i},{x}_{j}\right)=\phi \left({x}_{i}\right)}^{T} \phi \left({x}_{j}\right)$$

As a result of optimisation, the predicted class is calculated by summing all support vectors for samples within the margin. Where x is a given sample, α is the dual coefficient and equals zero for the samples outside the margin as follows:

3 $$\sum_{i \in SV}{y}_{i } {\alpha }_{i} K \left({x}_{i}, x\right)+b$$

C has an impact on the decision surface. SVM was trained by tuning C to balance between high value of C for correct classification and low value of for smooth decision surface.

The polynomial kernel is non-linear kernel calculated as follows:

4 $$K \left(x, x{\prime}\right)= {\left( 1+{x}^{T} {x}{\prime}\right)}^{d}$$

where d is the degree.

Gaussian kernel which is called Radial Basis Function (RBF) is a non-linear kernel calculated as follows:

5 $$K \left(x, x{\prime}\right)=\mathrm{exp}(- {\Vert x- {x}{\prime}\Vert }^{2}/2 {\sigma }^{2})$$

where σ is the standard deviation.

### Gradient boosting (GB) classifier

**Figure 5:**
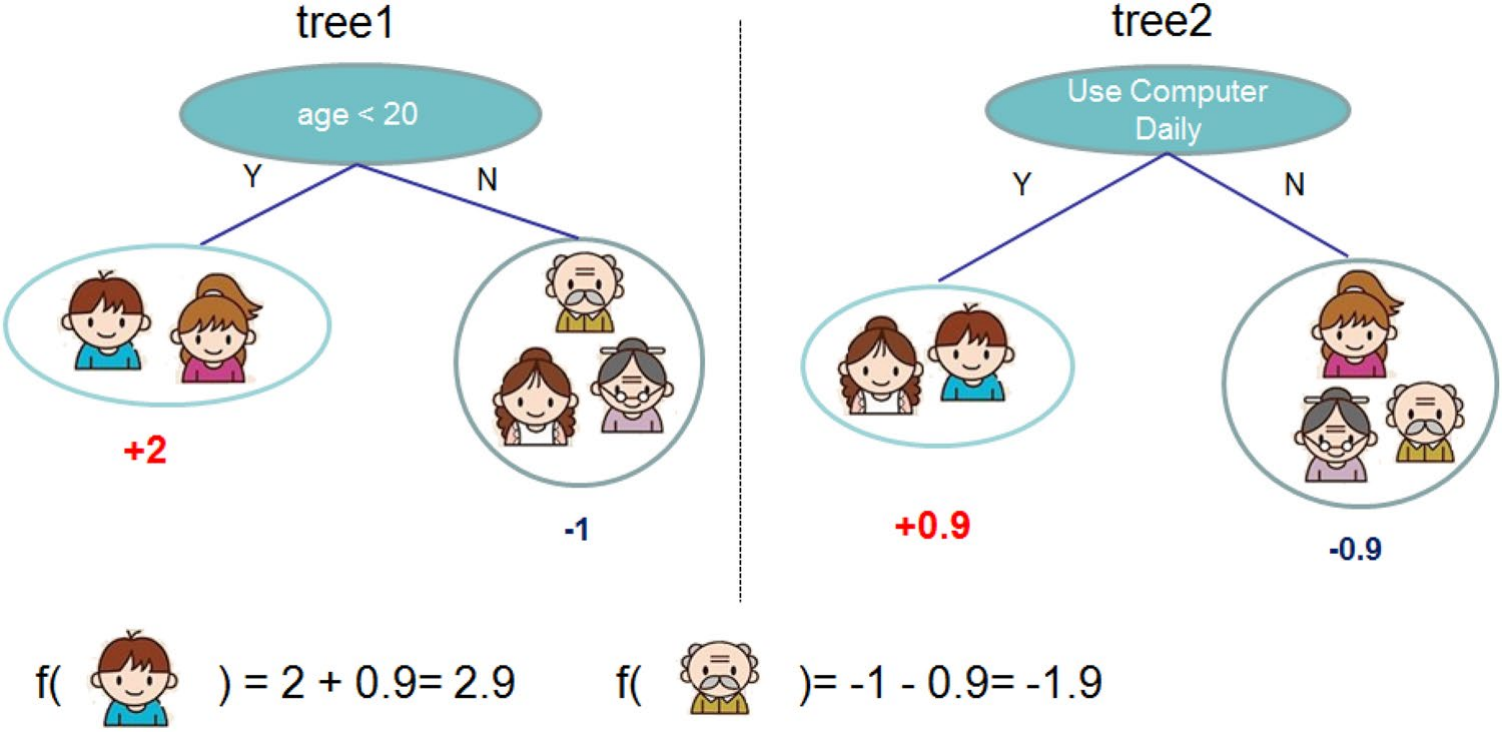
The structure and operation of GB (Hearst et al. 1998; Osman et al. 2021).

The Gradient boosting is another powerful model used in this work for the streamflow classification task. GB, a tree learning system, is based on an ensemble learning approach^42^. Figure 5 illustrates the structure of the gradient boosting classifier. The performance of GB is significantly impacted by the hyperparameters, such as learning rate, number of decision trees, and maximum depth. Thus, they need to be tuned carefully to find an optimal architecture and hyperparameters. Several experiments were conducted to evaluate the GB performance for the classification of streamflow values and to find the optimal hyperparameters. These optimal hyperparameters values can generate the best classification performance indicators in terms of F1 score and QWK^43,44^. It was found that GB with 200 number of trees, 0.01 learning rate, and max depth of 5 outperformed other GB models in terms of F1 score.

### Stacked ensemble

**Figure 6:**
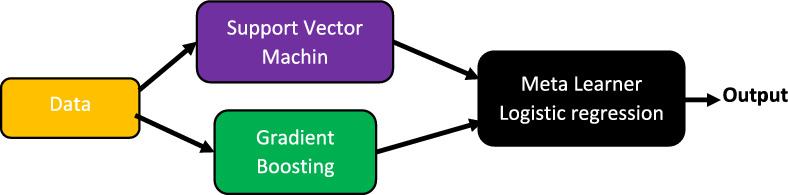
The structure of stacked ensemble.

The stacked ensemble is the third powerful model used in this work for the streamflow classification task. It is an ensemble learning method to find the optimal combination of a collection of classifiers using a stacking process. In order to get the optimum performance, the stacked ensemble also learns how to combine each of the classifiers^45^. This work investigated the stacked ensemble learning method, which employed a support vector machine and gradient boosting classifiers. The outputs of these classifiers were connected to the meta-learner of the logistic regression classifier to produce the final classification categories of streamflow. The structure of this stacked ensemble classifier is shown in Fig. 6.

### Long short-term memory (LSTM)

**Figure 7:**
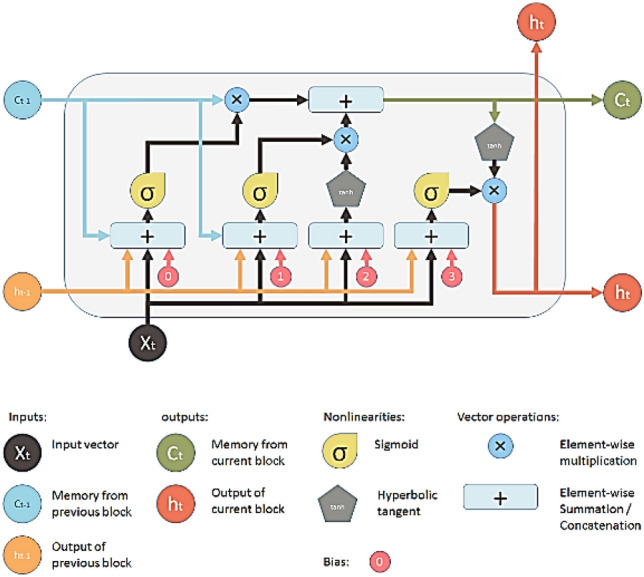
The structure LSTM neural network^47^.

The fourth effective model applied for the streamflow classification task was the Long short-term memory model. Recurrent Neural Networks (RNNs) are usually utilized for sequence modeling to capture temporal correlations^46^. LSTM is one of the RNNs to model the long-range sequences using a memory cell, as shown in Fig. 7, which acts as an accumulator of state information supported by control gates. LSTM structure has the advantage of overcoming the problem of gradient vanishing^47^. The parameters of LSTM were tuned to fit the data. Table 7 describes the architecture of LSTM. Figure 7 shows the structure of LSTM.

**Table 7:** Architecture of LSTM.

Layer (type)
Input Layer with 3 or 5 features (history of streamflow of 3- or 5-time steps)
LSTM with 512 nodes
Activation of RELU
Dropout of 0.4
Dense of 512 nodes
Activation of RELU
Dropout of 0.4
Dense of 10 nodes
Activation of SoftMax

The LSTM model was trained with training data using the following hyperparameters:

1. the learning rate was set to 0.001
2. the batch size was set to 32
3. the number of epochs was set to 100.
4. the loss function was categorical cross-entropy
5. the optimizer was Adam.

In summary, the previously developed models were used to classify the streamflow. The category of streamflow is affected by different factors, such as the history of streamflow values, as will be discussed in the section on experimental results. Each model was trained and evaluated to find the best architecture and hyperparameters for comparison stated in the section on experimental results. Table 8 compares used methods and shows Pros and Cons for each.

**Table 8:** Comparison between used methods to show Pros and Cons for each.

Method	Pros	Cons
SVM	Simple and efficient with small data	Not efficient with time series prediction
GB	High accuracy because it is based on ensemble learning	(1) More complex because it is based on ensemble learning (2) Not efficient with time series prediction
Stacked Ensemble learning	Higher accuracy than SVM and GB	More complex than SVM and GB
LSTM	Excellent for time series prediction	Requires big data to be efficient

### Performance metrics

The classification performance was evaluated using several metrics such as Accuracy, Precision, Recall, F1 score, Area Under Curve (AUC), and Quadratic Weighted kappa (QWK).

1. Accuracy is a metric that calculates number of correctly predicted samples over total samples.

6 $$\mathrm{Accuracy }=\frac{TP+TN}{TP+TN+FP+FN}$$

where TP: True Positive, TN: True Negative, FP: False Positive, FN: False Negative.

2. Precision (positive predictive value) is a metric to calculate the correctly identified positive samples over all predicted positive samples.

7 $$\mathrm{Precision}=\frac{TP}{TP+FP}$$

3. Recall (Sensitivity) is a measure that calculates correctly identified positive samples over all actual positive samples.

8 $$\mathrm{Recall }= \frac{TP}{TP+FN}$$

4. F1 score summarizes recall and precision in one metric.

9 $$\mathrm{F}1\mathrm{ score }= \frac{2 \times precision \times recall}{precision+recall}$$

5. Area Under Curve is a metric to show how much a classifier is robust with a varied threshold.

AUC is an area under receiver operating characteristic (ROC) curve that shows relation between false positive rate and true positive rate.

6. Quadratic Weighted kappa (QWK): Cohen's weighted kappa is a measure of agreement between observed rates, as shown in Table 9. A weighted Kappa is a metric to measure the similarity between predicted and actual values. An optimal score of 1.0 results from a complete match between predicted and actual values. The worst score, however, a -1, is the consequence of a significant difference between predicted and actual values. QWK considers the similarity between the classes beyond exclusively the class. This is suitable when ordinal or ranked variables are available, as presented in this work. The dataset used in this work has five or ten ratings that represent various streamflow value categories. The weight matrix that represents the difference between the ten categories in the ten classes scenario is shown in Table 10. The same concept can be applied to any number of classes.

**Table 9:** QWK interpretation.

QWK	Agreement
< 0	No
0.01–0.20	Slight
0.21–0.40	Fair
0.41–0.60	Moderate
0.61–0.80	Substantial
0.81–0.99	Almost perfect

**Table 10:** The Weight Matrix W represents the difference between the classes for ten classes scenario.

	Class1	Class2	Class3	Class4	Class5	Class6	Class7	Class8	Class9	Class10
Class1	0	0.0123	0.0494	0.1111	0.1975	0.3086	0.4444	0.6049	0.7901	1
Class2	0.0123	0	0.0123	0.0494	0.1111	0.1975	0.3086	0.4444	0.6049	0.7901
Class3	0.0494	0.0123	0	0.0123	0.0494	0.1111	0.1975	0.3086	0.4444	0.6049
Class4	0.1111	0.0494	0.0123	0	0.0123	0.0494	0.1111	0.1975	0.3086	0.4444
Class5	0.1975	0.1111	0.0494	0.0123	0	0.0123	0.0494	0.1111	0.1975	0.3086
Class6	0.3086	0.1975	0.1111	0.0494	0.0123	0	0.0123	0.0494	0.1111	0.1975
Class7	0.4444	0.3086	0.1975	0.1111	0.0494	0.0123	0	0.0123	0.0494	0.1111
Class8	0.6049	0.4444	0.3086	0.1975	0.1111	0.0494	0.0123	0	0.0123	0.0494
Class9	0.7901	0.6049	0.4444	0.3086	0.1975	0.1111	0.0494	0.0123	0	0.0123
Class10	1	0.7901	0.6049	0.4444	0.3086	0.1975	0.1111	0.0494	0.0123	0

We evaluated and compared the proposed models in this work using a bag of metrics. The training data was balanced because we used the balanced data method to select the ranges with the same number of samples in each class. However, classifiers evaluation and comparison were carried out using imbalanced testing data. Usually, accuracy is a proper metric to evaluate the performance of the classification model. However, accuracy has a drawback when the data is imbalanced and thus unable to evaluate performance in this work. Therefore, other evaluation metrics such as precision, recall, F1 score, AUC, and QWK were used. The larger values of these five metrics explain better data fitting and higher classification performance. The F1 score is considered an effective metric to measure classification performance with imbalanced data. The drawback of the F1 score is related to one fixed threshold used for classification. To address the previous limitation, AUC was another valuable metric utilized to highlight the robustness of the classification model with a varied threshold. Furthermore, a confusion matrix was also illustrated to show the details of four terms: true positive, true negative, false positive, and false negative.

### Experimental setup

The SVM, GB, and stacked ensemble models were trained on an Intel i7-5500U CPU using the scikit learn framework. The LSTM model, on the other hand, was developed on Google Collaboratory on K80 GPU with12 GB of RAM using the TensorFlow framework.

## Results and discussion

This section demonstrates various experiments carried out to train and evaluate several machine learning models, including support vector machine, gradient boosting, stacked ensemble, and long short-term memory. These experiments aim to evaluate models’ performance in terms of accuracy, precision, recall, F1 score, AUC, and QWK. In these experiments, the models’ hyperparameters were tuned to optimize the models and produce the best results. Two scenarios related to the number of categories were demonstrated: the streamflow values were divided into five categories in five class scenarios and ten categories in ten classes scenario. We aim to discover hidden patterns from the streamflow data for classification purposes.

### Support vector machine

The first set of experiments was conducted to demonstrate the impact of the history of previously observed streamflow to classify the future streamflow values one day ahead using a support vector machine. Using a balanced data method, we examined various values of history (number of previous days) in terms of F1 score, as shown in Tables 11 and 12 for five and ten categories, respectively. The maximum values are highlighted in bold font. The F1 scores were calculated considering the different history of streamflow values to predict one day ahead. The last one, three, five, seven, fifteen, or 30 days were evaluated to find the best F1 score of models in each river in each history value. It is clear that the scenario of 5 categories produced high performance in terms of maximum F1 scores of 81%, 84.0%, 82%, 75%, 62%, 80%, 66%, 80%, 73%, and 73% for Johor, Kedah, Kelantan, Melaka, N9, Pahang, Perak, Perlis, Selangor, Terengganu, respectively. On the other hand, due to the lack of data collected in WPKL, with only 365 samples for only one year, the F1 score is low at 37%. Additionally, the scenario of 10 categories produced a good performance in terms of maximum F1 scores of 66%, 69.0%, 64%, 60%, 65%, 56%, 61%, 58%, and 56% for Johor, Kedah, Kelantan, Melaka, Pahang, Perak, Perlis, Selangor, Terengganu, respectively. On the other hand, due to the lack of data collected in WPKL, with only 365 samples for only one year, the F1 score is low at 17%. Furthermore, the annual variation of N9 illustrates a small range of streamflow and the inability of SVM to capture any pattern in the N9 river’s stream data, resulting in a low F1 score of 34%.

**Table 11:** F1 score of SVM for five categories with balanced data method for various previous days.

F1 score				
River\History	1	**3**	**5**	7
Johor	0.59	**0.66**	**0.66**	0.65
Kedah	0.66	**0.69**	**0.69**	0.67
Kelantan	0.61	**0.64**	**0.64**	0.63
Melaka	**0.6**	**0.6**	0.59	0.56
N9	0.33	**0.34**	0.32	0.32
Pahang	0.58	**0.65**	0.64	0.64
Perak	**0.56**	0.54	**0.56**	0.53
Perlis	**0.61**	0.6	0.54	0.55
Selangor	0.56	**0.58**	0.57	**0.58**
Terengganu	**0.56**	**0.56**	**0.56**	0.55
WPKL	0.16	**0.17**	0.14	0.12

**Table 12:** F1 score of SVM for ten categories with balanced data method for various previous days.

F1-score						
River/history	1	3	5	7	15	30
Johor	0.77	**0.81**	**0.81**	0.8	0.79	0.74
Kedah	0.82	**0.84**	0.83	0.83	0.81	0.78
Kelantan	0.8	**0.82**	**0.82**	0.81	0.8	0.78
Melaka	0.74	**0.75**	0.74	0.73	0.7	0.64
N9	**0.62**	0.59	0.57	0.56	0.51	0.45
Pahang	0.76	**0.8**	**0.8**	**0.8**	0.77	0.72
Perak	**0.66**	0.65	0.65	0.62	0.62	0.55
Perlis	**0.8**	0.77	0.78	0.73	0.68	0.61
Selangor	0.72	**0.73**	**0.73**	**0.73**	0.71	0.68
Terengganu	0.72	**0.73**	0.72	0.72	0.71	0.69
WPKL	0.27	0.32	**0.37**	0.28	0.22	0.24

The metrics of SVM, including average accuracy, average recall, average precision, and average F1 score, were calculated for each river data in two scenarios of five classes and ten classes using the balanced data method. In this method, the training samples were distributed evenly between all categories. However, testing data were imbalanced. The metrics shown in Tables 13 and 14 were found for the best model selected according to the maximum F1 score reported in Tables 11 and 12. The empty cells in the AUC column resulted from the unavailability of all classes in testing data, even if they are available in training data.

**Table 13:** Classification report of SVM for five categories with balanced data method.

	Avg Accuracy	Avg Recall	Avg Precision	Avg F1 score	Avg AUC
Johor	0.65	0.66	0.66	0.66	0.95
Kedah	0.7	0.69	0.69	0.69	0.95
Kelantan	0.64	0.65	0.63	0.64	0.95
Melaka	0.59	0.62	0.6	0.60	0.92
N9	0.43	0.38	0.44	0.34	0.82
Pahang	0.69	0.65	0.65	0.65	0.95
Perak	0.67	0.54	0.58	0.56	–
Perlis	0.83	0.6	0.61	0.61	0.8
Selangor	0.64	0.59	0.59	0.58	0.91
Terengganu	0.6	0.57	0.57	0.56	0.91
WPKL	0.31	0.2	0.22	0.17	-

**Table 14:** Classification report of SVM for ten categories with balanced data method.

	Avg Accuracy	Avg Recall	Avg Precision	Avg F1 score	Avg AUC
Johor	0.8	0.8	0.81	0.81	0.96
Kedah	0.83	0.84	0.84	0.84	0.97
Kelantan	0.83	0.83	0.82	0.82	0.97
Melaka	0.74	0.76	0.76	0.75	0.93
N9	0.64	0.62	0.63	0.62	0.84
Pahang	0.83	0.8	0.8	0.80	0.97
Perak	0.74	0.66	0.69	0.66	0.93
Perlis	0.87	0.8	0.8	0.80	0.87
Selangor	0.74	0.73	0.74	0.73	0.92
Terengganu	0.76	0.73	0.72	0.73	0.93
WPKL	0.7	0.39	0.43	0.37	0.57

As discussed earlier, the testing data were imbalanced even though the training data were balanced in the balanced data method. Therefore, accuracy alone is not enough to measure model performance; thus, the F1 score was calculated. Additionally, as well known in machine learning classification methods, an increasing number of categories leads to more complex classification and lower F1 scores.

### Gradient boosting

The second set of experiments used gradient boosting to illustrate the influence of previously observed streamflow on classifying the predicted streamflow values one day ahead. We compared various values of history (number of previous days) in terms of F1 score, as shown in Tables 15 and 16 for five and ten categories, respectively, with a balanced data method. The maximum values are highlighted in bold font. The F1 scores were calculated considering the different history of streamflow values to predict one day ahead. The last one, three, five, seven, or fifteen days were evaluated to find the best F1 score of models in each river in each history value. The scenario of 5 categories produced high performance in terms of maximum F1 scores of 80%, 83.0%, 82%, 78%, 64%, 79%, 67%, 80%, 76%, and 74% for Johor, Kedah, Kelantan, Melaka, N9, Pahang, Perak, Perlis, Selangor, Terengganu, respectively. On the other hand, as we mentioned in SVM, the small number of samples collected in WPKL (365 samples) was behind the low F1 score (34%). Additionally, the scenario of 10 categories produced a good performance in terms of maximum F1 scores of 64%, 68.0%, 62%, 60%, 61%, 57%, 63%, 58%, and 58% for Johor, Kedah, Kelantan, Melaka, Pahang, Perak, Perlis, Selangor, Terengganu, respectively. However, the poor F1 score (19%) in WPKL was due to only 365 samples used. The same explanation for the annual variation of N9 from SVM also applied to GB. Due to the narrow range of streamflow in the N9 river, GB was unable to identify the patterns, and as a result, the F1 score was low (47%).

**Table 15:** F1 score of GB for five categories with balanced data method for various previous days.

F1 score				
River/history	1	3	5	7
Johor	0.76	**0.8**	**0.8**	**0.8**
Kedah	0.82	**0.83**	**0.83**	**0.83**
Kelantan	0.8	0.81	0.81	0.81
Melaka	0.77	**0.78**	**0.78**	**0.78**
N9	**0.64**	0.63	**0.64**	0.63
Pahang	0.76	**0.79**	**0.79**	**0.79**
Perak	0.65	**0.67**	0.65	0.65
Perlis	**0.8**	0.77	0.79	0.78
Selangor	0.75	**0.76**	**0.76**	**0.76**
Terengganu	0.72	0.72	0.73	0.73
WPKL	0.24	**0.34**	0.33	0.28

**Table 16:** F1 score of GB for ten categories with balanced data method for various previous days.

F1 score				
River/history	1	3	5	7
Johor	0.59	0.63	**0.64**	**0.64**
Kedah	0.67	**0.68**	**0.68**	**0.68**
Kelantan	0.61	0.61	0.61	0.61
Melaka	**0.60**	0.54	0.52	0.55
N9	**0.47**	0.46	0.46	0.45
Pahang	0.57	**0.61**	**0.61**	**0.61**
Perak	**0.57**	0.54	0.55	0.55
Perlis	0.61	0.58	0.61	0.6
Selangor	0.56	**0.58**	0.57	0.57
Terengganu	0.57	0.57	0.56	0.57
WPKL	0.14	0.16	**0.19**	0.16

Tables 17 and 18 show the classification report of GB for five and ten categories scenarios, respectively, with a balanced data method. In this method, the training samples were distributed evenly between all categories. Testing data, though, were imbalanced. For each river dataset, the macro average of precision, recall, and F1 score, as well as the average accuracy, were computed. The empty cells in the AUC column resulted from the unavailability of all classes in testing data even though they are available in training data. From previous classification reports, it can be deduced that the performance of the GB model with a balanced data method was high; thus, the GB was able to learn patterns from observed streamflow values.

**Table 17:** Classification report of GB for five categories with balanced data method.

	Avg Accuracy	Avg Recall	Avg Precision	Avg F1 score	Avg AUC
Johor	0.79	0.8	0.8	0.8	0.95
Kedah	0.82	0.83	0.83	0.83	0.97
Kelantan	0.82	0.83	0.8	0.82	0.97
Melaka	0.76	0.79	0.78	0.78	0.94
N9	0.67	0.64	0.64	0.64	0.87
Pahang	0.82	0.79	0.79	0.79	0.96
Perak	0.74	0.65	0.71	0.67	0.93
Perlis	0.87	0.8	0.8	0.8	0.88
Selangor	0.78	0.76	0.76	0.76	0.93
Terengganu	0.78	0.74	0.74	0.74	0.93
WPKL	0.69	0.34	0.36	0.34	0.56

**Table 18:** Classification report of GB for ten categories with balanced data method.

	Avg Accuracy	Avg Recall	Avg Precision	Avg F1 score	Avg AUC
Johor	0.63	0.64	0.64	0.64	0.94
Kedah	0.69	0.69	0.68	0.68	0.95
Kelantan	0.64	0.65	0.61	0.62	0.95
Melaka	0.6	0.61	0.6	0.60	0.92
N9	0.5	0.47	0.48	0.47	0.83
Pahang	0.66	0.61	0.62	0.61	0.94
Perak	0.68	0.57	0.59	0.57	–
Perlis	0.84	0.62	0.64	0.63	0.81
Selangor	0.63	0.58	0.58	0.58	0.91
Terengganu	0.62	0.58	0.59	0.58	0.92
WPKL	0.48	0.2	0.18	0.19	–

### Stacked ensemble

We carried out the third set of experiments to demonstrate the impact of the history of previously observed streamflow to classify the future streamflow values one day ahead in each river using a stacked ensemble. With a balanced data method, SVM, GB, and stacked ensemble were compared in terms of F1 score as shown in Table 19 for ten categories scenario. The best F1 values regarding various histories of streamflow for each river are highlighted in bold font. Stacked ensemble shows higher performance than SVM and GB in terms of F1 score in a scenario of 10 categories with 67%, 69%, 64%, 61%, 48%, 60%, 59%, and 59% for Johor, Kedah, Kelantan, Melaka, N9, Perak, Selangor, Terengganu, respectively.

**Table 19:** Comparison between SVM, GB, and stacked ensemble for ten categories with balanced data method.

F1 score	SVM	XGB	Ensemble
Johor	0.66	0.64	**0.67**
Kedah	0.69	0.68	**0.69**
Kelantan	0.64	0.62	**0.64**
Melaka	0.60	0.60	**0.61**
N9	0.34	0.47	**0.48**
Pahang	**0.65**	0.61	0.64
Perak	0.56	0.57	**0.60**
Perlis	0.61	**0.63**	0.61
Selangor	0.58	0.58	**0.59**
Terengganu	0.56	0.58	**0.59**

The QWK for the stacked ensemble was calculated for ten categories with a data balanced method, as shown in Table 20. The values of QWK were more than 0.82 in all rivers except N9 and WPKL. The high values of QWK (> 0.82) refer to almost perfect agreement between actual and predicted classes. The high QWK evaluates the similarity between the classes in addition to the class. The 0.807 QWK in N9 refers to a substantial agreement, and the 0.31 QWK in WPKL refers to a fair agreement. On the other hand, the values of QWK for several rivers, such as Johor, Kedah, Kelantan, and Pahang, are more than 95% which means the superior performance of the stacked ensemble and its ability to learn informative patterns from streamflow data available in these rivers.

**Table 20:** QWK for stacked ensemble for ten categories with balanced data method.

	QWK	Agreement
Johor	0.95	Almost perfect
Kedah	0.96	Almost perfect
Kelantan	0.95	Almost perfect
Melaka	0.90	Almost perfect
N9	0.807	Substantial
Pahang	0.97	Almost perfect
Perak	0.91	Almost perfect
Perlis	0.84	Almost perfect
Selangor	0.94	Almost perfect
Terengganu	0.94	Almost perfect
WPKL	0.31	Fair

**Figure 8:**
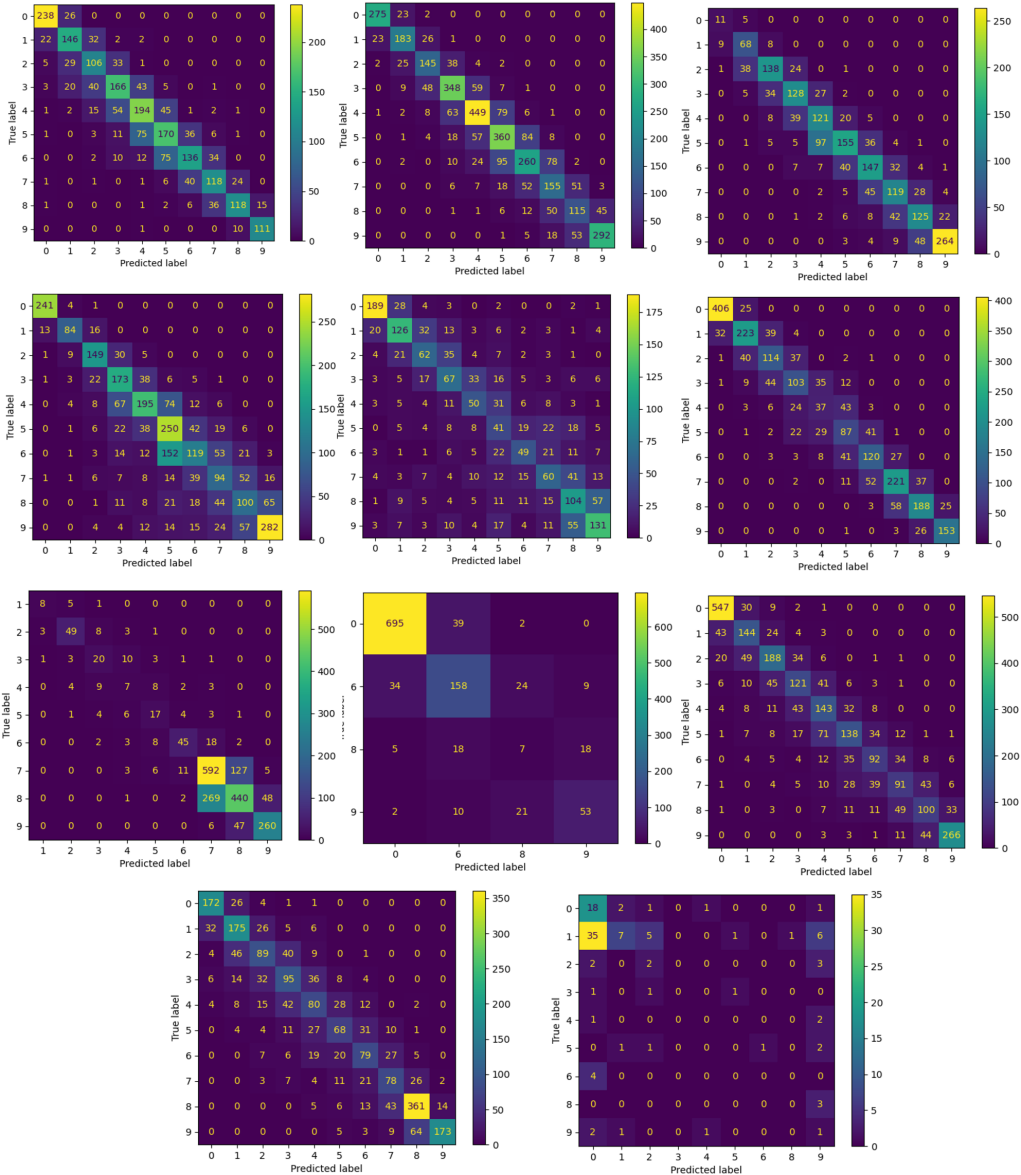
Confusion matrix for each river using stacked ensemble for ten classes scenario for Johor, Kedah, Kelantan, Melaka, N9, Pahang, Perak, Perlis, Selangor, Terengganu, WPKL ordered from eft to right and from top to down.

Figure 8 shows the confusion matrix for each river using a stacked ensemble for ten classes scenario. The high capability of the stacked ensemble to classify the streamflow values are so clear from these confusion matrixes. Since the categories in the streamflow prediction task are ordinal, QWK can be an appropriate metric to measure the model's success in classifying data.. The misclassification in this model occurred simply by predicting the incorrect class, which was so close to the actual one. As mentioned before, the testing data were imbalanced, as seen in the confusion matrixes in Fig. 8. Due to the limited streamflow classes in the testing data, Perils river showed only four outputs. The poor findings in WPL are due to a dearth of data from this river.

### Long short-term memory

The results of the fourth set of experiments demonstrated how the LSTM classified the future streamflow one day in advance, given the history of previously observed streamflow. According to Table 21, we utilized the data balanced method to compare various historical streamflow values based on the F1 score for the ten categories to predict one, three and five days ahead. The ten categories scenario yielded strong results, with maximum F1 scores of 66%, 69.0%, 64%, 61%, 63%, 59%, 62%, 60%, and 57% for Johor, Kedah, Kelantan, Melaka, Pahang, Perak, Perlis, Selangor, Terengganu, respectively. In contrast, the poor F1 score (16%) in WPKL was owing to the limited 365 samples. Due to a narrow range of streamflow in the N9 river, which is also the cause for the annual variation of N9, LSTM was unable to capture the patterns, and thus F1 score was low (47%).

**Table 21:** F1 score of LSTM for ten categories with balanced data method for various previous days.

River/history	1	3	5
Johor	0.58	**0.66**	**0.66**
Kedah	0.67	0.68	**0.69**
Kelantan	0.62	**0.64**	0.63
Melaka	**0.61**	0.57	0.58
N9	0.39	0.46	**0.47**
Pahang	0.58	**0.63**	0.60
Perak	0.56	0.54	**0.59**
Perlis	**0.62**	**0.62**	0.60
Selangor	0.57	**0.60**	0.58
Terengganu	**0.57**	0.56	0.56
WPKL	0.13	**0.16**	0.06

Significant values are in bold.

The QWK for long short-term memory was calculated for ten categories with a data balanced method, as shown in Table 22. The values of QWK were more than 0.82 in all rivers except N9 and WPKL. The high values of QWK (> 0.82) referred to almost perfect agreement between actual and predicted classes. The 0.79 QWK in N9 denoted to a substantial agreement, but the 0.35 QWK in WPKL implied a fair agreement.

**Table 22:** QWK for LSTM for ten categories with balanced data method.

	QWK	Agreement
Johor	0.95	Almost perfect
Kedah	0.96	Almost perfect
Kelantan	0.95	Almost perfect
Melaka	0.90	Almost perfect
N9	0.79	Substantial
Pahang	0.97	Almost perfect
Perak	0.91	Almost perfect
Perlis	0.84	Almost perfect
Selangor	0.95	Almost perfect
Terengganu	0.94	Almost perfect
WPKL	0.35	Fair

### Classification of few days ahead

We added another experiment to explore the capability of a stacked ensemble to generalize and learn new patterns to predict the category of few days ahead. The F1 score of category prediction one-to-three-time steps ahead (days) is shown in Table 23. It is clear that the category prediction of the streamflow one day ahead of SF + 1 outperformed the prediction of the streamflow two days or three days ahead (SF + 2 and SF + 3) in terms of F1 score and QWK. Table 24 shows QWK to predict various days ahead. It is clear that the F1 score and QWK for predictions of 3 days ahead are not high because of complex hidden patterns that are not easy to be discovered for the n days ahead prediction task.

**Table 23:** F1 score of stacked ensemble for ten categories scenario to classify several days ahead.

F1 score	Ensemble		
N day ahead	t + 1	t + 2	t + 3
Johor	0.67	0.46	0.36
Kedah	0.69	0.57	0.48
Kelantan	0.64	0.50	0.40
Melaka	0.61	0.48	0.42
N9	0.48	0.32	0.28
Pahang	0.64	0.44	0.34
Perak	0.60	0.49	0.41
Perlis	0.61	0.52	0.44
Selangor	0.59	0.42	0.35
Terengganu	0.59	0.48	0.41

**Table 24:** QWK of stacked ensemble for ten categories scenario to classify several days ahead.

QWK	Ensemble		
N day ahead	t + 1	t + 2	t + 3
Johor	0.95	0.86	0.77
Kedah	0.96	0.90	0.87
Kelantan	0.95	0.89	0.84
Melaka	0.90	0.83	0.78
N9	0.807	0.68	0.62
Pahang	0.97	0.92	0.87
Perak	0.91	0.86	0.84
Perlis	0.84	0.69	0.58
Selangor	0.94	0.87	0.83
Terengganu	0.94	0.91	0.88

### Comparison between models for streamflow classification

**Figure 9:**
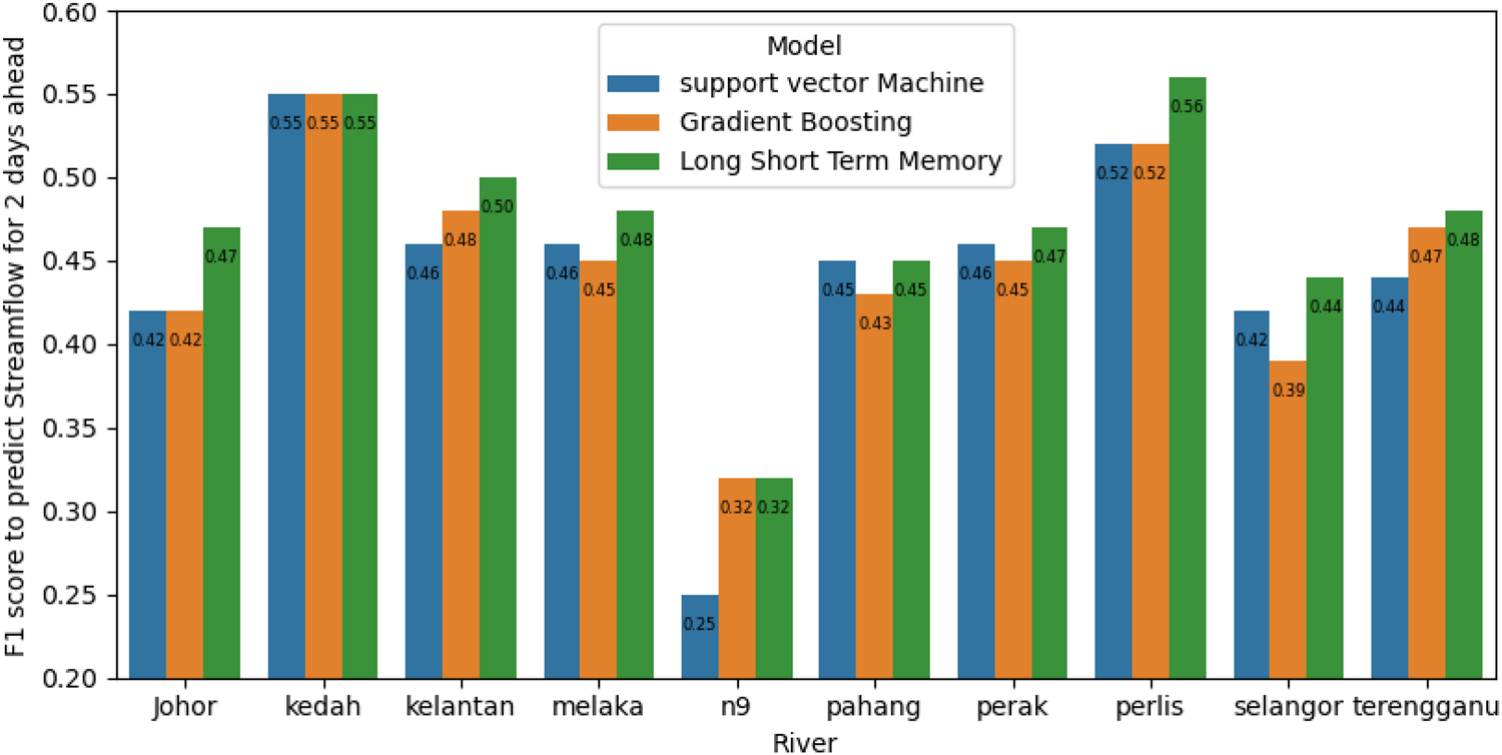
Comparison between GB, SVM, and LSTM in terms of F1 score for classification of 2 days ahead in ten classes scenario.

**Figure 10:**
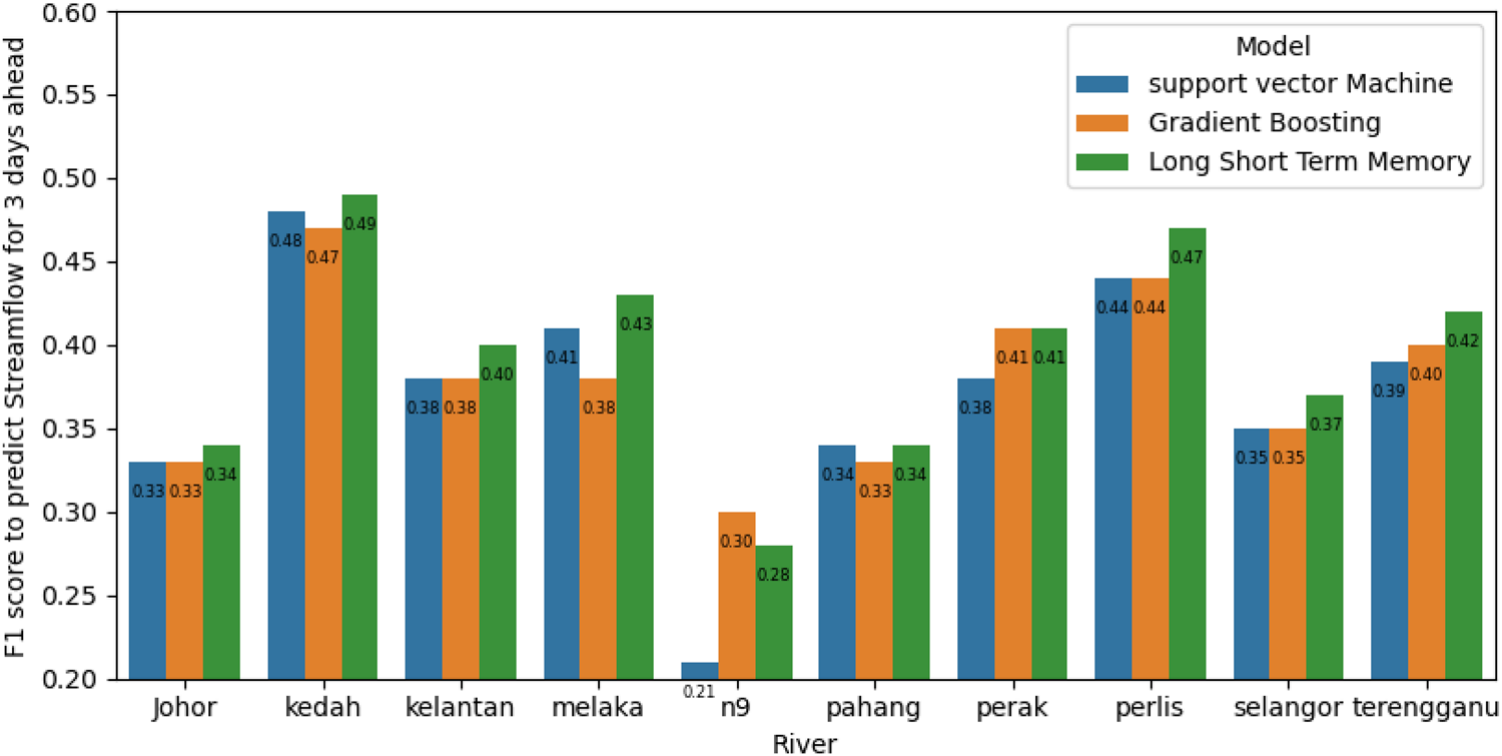
Comparison between GB, SVM, and LSTM in terms of F1 score for classification of 3 days ahead in ten classes scenario.

Figures 9 and 10 depict the F1 score GB, SVM, and LSTM two and three days ahead of classification, respectively. In the scenario of ten categories for most rivers, including Johor, Kelantan, Melaka, Perak, Perlis, Selangor, and Terengganu, it was discovered that LSTM outperformed GB and SVM. It is crucial to act proactively to avoid risks earlier owing to the model’s capacity to anticipate streamflow class for n ahead days. The LSTM was able to learn the mapping between streamflow time series of 2 or 3 days ahead more than SVM and GB. The performance of SVM and GB is different from one river to another. In other words, in some rivers, SVM outperformed GB, and in others, GB surpassed SVM in terms of F1 score.

In summary, the findings in this paper are summarized as follows:

1. The streamflow prediction was formulated as a time series classification with discrete ranges of values, each representing a class to classify streamflow into five or ten.
2. Prediction of classes into five categories is more accurate than prediction of 10 categories.
3. LSTM outperformed others in predicting n-time steps of streamflow because LSTM is able to learn the mapping between streamflow time series of 2 or 3 days ahead more than support vector machine (SVM) and gradient boosting (GB).
4. Stacked ensemble learning of the SVM and GB achieved higher performance than SVM and GB in terms of F1 score and quadratic weighted kappa.

## Conclusion and future work

An investigation of streamflow regression as a classification machine learning approach has been described. Two scenarios-based streamflow classifications were evaluated using four AI-based techniques, namely, SVM, GB, and LSTM, an ensemble stacking model in the majority of the main rivers in Malaysia. Forecasting multiple rivers is essential as it provides spatial forecast information for efficient basin-wide reservoir management. The findings demonstrated that, despite having been used to solve a streamflow classification problem, LSTM's memory-storing capabilities allow it to extract the temporal pattern from the streamflow time series, as evidenced by the highest F1 score in all the selected rivers. In addition, this work's findings could be exploited in any situation where a time series regression is to be transitioned to classification, provided that the forecast outputs are deterministic or mechanical (e.g., reservoir operation). The limitation in this streamflow prediction task is related to uncertainty and complex hidden patterns available in each river. These patterns should be extracted well to produce high performance and accuracy. This leads to inability to build one predictive model for modelling all rivers at the same time. In other words, each river requires a specific predictive model that is able to fit its own patterns. For future works, we intend to explore recent attention based deep learning models after collecting more streamflow data to improve the prediction accuracy. The impact of dam construction on regional precipitation has been investigated in the literature confirming the correlation between dam construction and regional precipitation^48^. This correlation study can be useful in our future study to explore the correlation between the dam construction and streamflow level categories which plays a significant role to plan the water resources.

## Data Availability

The finding data of this study is available from the corresponding author upon request.

## Code availability

All code used in this study is available from the corresponding author upon request.
